# Difficulties with Speech-in-Noise Perception Related to Fundamental Grouping Processes in Auditory Cortex

**DOI:** 10.1093/cercor/bhaa311

**Published:** 2020-11-02

**Authors:** Emma Holmes, Peter Zeidman, Karl J Friston, Timothy D Griffiths

**Affiliations:** Wellcome Centre for Human Neuroimaging, UCL Queen Square Institute of Neurology, UCL, London WC1N 3AR, UK; Wellcome Centre for Human Neuroimaging, UCL Queen Square Institute of Neurology, UCL, London WC1N 3AR, UK; Wellcome Centre for Human Neuroimaging, UCL Queen Square Institute of Neurology, UCL, London WC1N 3AR, UK; Wellcome Centre for Human Neuroimaging, UCL Queen Square Institute of Neurology, UCL, London WC1N 3AR, UK; Biosciences Institute, Faculty of Medical Sciences, Newcastle University, Newcastle upon Tyne NE2 4HH, UK

**Keywords:** auditory scene analysis, DCM, figure-ground, fMRI, speech perception

## Abstract

In our everyday lives, we are often required to follow a conversation when background noise is present (“speech-in-noise” [SPIN] perception). SPIN perception varies widely—and people who are worse at SPIN perception are also worse at fundamental auditory grouping, as assessed by figure-ground tasks. Here, we examined the cortical processes that link difficulties with SPIN perception to difficulties with figure-ground perception using functional magnetic resonance imaging. We found strong evidence that the earliest stages of the auditory cortical hierarchy (left core and belt areas) are similarly disinhibited when SPIN and figure-ground tasks are more difficult (i.e., at target-to-masker ratios corresponding to 60% rather than 90% performance)—consistent with increased cortical gain at lower levels of the auditory hierarchy. Overall, our results reveal a common neural substrate for these basic (figure-ground) and naturally relevant (SPIN) tasks—which provides a common computational basis for the link between SPIN perception and fundamental auditory grouping.

## Introduction

One of the greatest challenges of everyday listening is the requirement to understand speech when background noise is present (“speech-in-noise” [SPIN] perception). Unlike speech perception in quiet, listeners must segregate speech from other sounds. Yet, we do not fully understand the processes involved in segregating speech from background noise, and how these relate to grouping processes engaged by more basic auditory tasks. Here, we used functional magnetic resonance imaging (fMRI) to examine the common neural correlates for grouping nonlinguistic auditory stimuli and for grouping speech in background noise.

Nonlinguistic grouping can be assessed using an auditory figure-ground paradigm that tests the ability to track pure tones that retain the same frequency over time (the “figure”) among a “background” of tones of random frequencies (Teki et al. 2011, 2013). Holmes and Griffiths (2019) found that individual differences in figure-ground perception covaried with in SPIN perception, showing that these tasks are inherently linked. Similar to SPIN perception, the “figure” and “background” tones used by Holmes and Griffiths (2019) overlap in frequency, so figures cannot be detected through spectral separation. Instead, this may be achieved by detecting temporal coherence between figure elements, as measured by cross correlation (Shamma et al. 2011; Teki et al. 2013).

The site of grouping processes that permit distinct representations of auditory figure and ground is currently unclear. From first principles, auditory figure-ground analysis requires a mechanism that can integrate information across different frequency channels. Although single-frequency tuning is common in auditory cortex neurons, primate recording studies have also demonstrated neurons tuned to multiple frequencies, even in primary auditory cortex (Rauschecker 1998; Elhilali, Ma, et al. 2009a; Elhilali, Xiang, et al. 2009b; Wang 2013). Nonhuman primate fMRI has demonstrated ensemble responses to segregated figures in high-level auditory cortex (belt and parabelt) (Schneider et al. 2018). Human fMRI (Teki et al. 2011) and MEG (Teki et al. 2016) studies also implicate high-level auditory cortex and the intraparietal sulcus (IPS) in figure representation. However, these studies all used an irrelevant (e.g., visual) task—whereas the behavioral relationship between figure-ground and SPIN perception (Holmes and Griffiths 2019) was based on active task performance. Figure-ground perception that is active and challenging might engage primary auditory cortex to a greater extent—as suggested by a MEG study of figure-ground perception (Molloy et al. 2019), which showed effects of attentional load on left human primary cortex.

In the present fMRI study, we were specifically interested in brain regions that are similarly engaged by active figure-ground analysis and SPIN perception. Previous studies of SPIN perception have revealed responses in a wide variety of areas, which—broadly speaking—appear to overlap with parts of auditory cortex that have been associated with figure-ground perception. A meta-analysis by Adank (2012) shows consistent activation in bilateral STS slightly anterior to Heschl’s gyrus under difficult listening conditions, which appears to overlap with STS activity reported by Teki et al. (2011) in response to figure-ground salience and also with widespread activity in auditory cortex reported by Molloy et al. (2019). A separate meta-analysis by Alain et al. (2018) found that challenging speech perception (e.g., speech at a lower target-to-masker ratio [TMR] or spectrally degraded speech) was associated with consistent activity in bilateral STG and insula. SPIN perception, specifically, was associated with activity in the left inferior parietal lobule. However, the co-ordinates in left IPS reported for figure-ground perception (Teki et al. 2011) are either more superior or more posterior and medial to the peak co-ordinates in the left inferior parietal lobule reported by Alain et al. (2018); therefore, we predict less overlap in parietal areas. Primary (core; Te1.0) auditory cortex has typically been absent in studies comparing different SPIN conditions, but it shows a relationship with the intelligibility of speech presented alone: it shows greater activity for clear relative to degraded (vocoded) speech, greater activity for degraded than unintelligible speech, and greater activity when the intelligibility of degraded speech is enhanced by presenting a matching word prime (Wild et al. 2012). Putative belt areas of auditory cortex show similar patterns: Anterolateral Heschl’s gyrus (roughly corresponding to Te1.2) shows a preference for clear compared with vocoded speech (Nourski et al. 2019), and posterior Heschl’s gyrus (roughly corresponding to Te1.1) shows a relationship with speech intelligibility in signal-correlated noise (Davis et al. 2011). Beyond auditory cortex, SPIN tasks have been shown to engage fronto-parietal areas (Hill and Miller 2010; Eckert et al. 2016) that are commonly associated with attention—in particular, the inferior frontal gyrus (Alain et al. 2018), but also the inferior frontal sulcus and middle frontal gyrus (Binder et al. 2004; Scott et al. 2004; Zekveld et al. 2006; Davis et al. 2011). Activity has also been observed in cingulo-opercular regions, including the insula, frontal operculum, and cingulate gyrus (Eckert et al. 2016; Vaden Jr et al. 2013, 2016).

Here, we used a within-subjects design to reveal areas that were more engaged in figure-ground and SPIN perception when they were more challenging. Comparing different levels of acoustic challenge within each task allowed us to focus on the areas associated with the difficulty involved in perceiving figures among background tones and speech against noise, rather than areas associated with similarities or differences in the acoustic properties of figure-ground and SPIN stimuli. We compared brain activity (here, estimated using blood-oxygen level-dependent [BOLD] activity) during active figure-ground analysis and SPIN perception, at different TMRs, and modeled responses in the auditory cortical hierarchy to establish the mechanism by which these effects operate. To ensure that behavioral performance did not explain differences between the two tasks, we selected TMRs for which accuracy was matched. To assess the functional anatomy of the shared behavioral relationship, we selected two different TMRs—which, here, correspond to the salience of the figure and speech or, in other words, the difficulty of the tasks—that led to different performance levels.

Given we were interested in commonalities between the two tasks, we adopted a Bayesian analysis—specifically, we used dynamic causal modeling (DCM), which estimates effective connectivity (i.e., directed neuronal coupling). An advantage of using DCM is that it allowed us to compare—using Bayesian model comparison—models in which task-specific effects of difficulty are present or absent. This overcomes a common limitation of classical (frequentist) statistics that “failure to reject the null” is not evidence for no difference. In contrast, using DCM allows us to explicitly compare models that include only commonalities (i.e., main effect of difficulty) and models that also include effects that are specific to SPIN or figure-ground tasks (i.e., task-difficulty interactions). If models that include only commonalities have greater evidence than those that also include effects specific to figure-ground or SPIN tasks, we can confidently conclude that greater difficulty in both tasks is mediated by similar changes in directed neuronal coupling.

Based on the aforementioned studies, we anticipated that the greatest overlap of functional integration between figure-ground and SPIN perception would occur in auditory cortex. Regarding standard univariate BOLD analyses, we predicted the two tasks would activate distinct higher level regions beyond auditory cortex (e.g., IPS, IFG, and cingulo-opercular regions), but we predicted overlapping activations in early auditory cortex. A whole brain analysis confirmed that the greatest area of overlap was indeed in auditory cortex. Therefore, for the DCM analysis, we focussed on subregions of auditory cortex—and assessed effective connectivity within and between these subregions. We might have expected to find common top-down effective connectivity to primary cortex, perhaps reflecting greater reliance on prior expectations when grouping is challenging (e.g., Marslen-Wilson and Tyler 1980; McClelland and Elman 1986; Norris and Mcqueen 2008; Mattys et al. 2012; Norris et al. 2015; Peelle 2018). However, we found evidence for a model in which common effects occur at the level of primary auditory cortex, consistent with the active component of these tasks (Molloy et al. 2019).

## Materials and Methods

### Subjects

Forty-nine participants completed the experiment. We measured their pure-tone audiometric thresholds at octave frequencies between 0.25 and 8 kHz in accordance with BS EN ISO 8253-1 (British Society of Audiology 2004). We excluded one participant who had a mild sloping hearing loss; all other participants had 6-frequency average thresholds better than 20 dB HL in either ear. We also excluded 4 participants who were not native English speakers, leaving 44 participants—which is the number we aimed to analyse based on an a priori power analysis. A sample size of 44 was estimated using NeuroPower (http://neuropowertools.org) with power = 0.8, and was based on publicly available fMRI data reported by Hakonen et al. (2017); https://identifiers.org/neurovault.collection:1626. The 44 participants (23 male) we tested were 19–35 years old (median = 22.7 years; interquartile range = 6.0) and all reported that they were right-handed.

The study was approved by the University College London Research Ethics Committee, and was performed in accordance with relevant guidelines and regulations. Informed consent was obtained from all participants.

### Stimuli

Stochastic figure-ground (SFG) stimuli were based on Holmes and Griffiths (2019). They contained 50-ms chords, gated by a 10-ms raised-cosine ramp, with 0 ms interchord interval. Each chord contained multiple pure tones at frequencies selected from a logarithmic scale between 179 and 7246 Hz (1/24th octave separation). The stimuli contained a figure that lasted 2100 ms (42 chords) and a background that lasted 3100 ms (62 chords). The background comprised 5–15 pure tones, whose frequencies were selected randomly at each time window. The figure comprised 3 components; the frequencies were selected randomly on each trial and were the same for the entire figure duration. The figure began 500 ms (10 chords) after the background. For half of stimuli, 4 chords (lasting 200 ms) were omitted from the figure. For the other half, the same number of components (3) were omitted from the background (4 chords; 200 ms). The omitted components began 20–42 chords after the onset of the figure-ground stimulus (10–32 chords after the onset of the figure); the components were always omitted while the figure was present, even if they were omitted from the background.

Sentences for the SPIN task were from the English version of the Oldenburg matrix set and were recorded by a male native-English speaker with a British accent. The sentences are of the form “<Name> <verb> <number> <adjective> <noun>” and contain 10 options for each word (see Table 1). An example is “Rachel brought four large chairs”. Recorded sentences were normalized to the same root-mean-square amplitude and lasted on average 2.2 s (standard deviation = 0.1). The sentences were presented simultaneously with 16-talker babble (all male talkers), which began 500 ms before the sentence began, ended 500 ms after the sentence ended, and was gated by a 10-ms raised-cosine ramp. The babble was taken from a continuous track lasting 20 s; a different segment of the babble was selected on each trial.

**Table 1:** Words from the English version of the Oldenburg International Matrix corpus, which was used in the SPIN task

Name	Verb	Number	Adjective	Noun
Alan	Got	Three	Large	Desks
Doris	Sees	Nine	Small	Chairs
Kathy	Brought	Seven	Old	Tables
Lucy	Gives	Eight	Dark	Toys
Nina	Sold	Four	Heavy	Spoons
Peter	Prefers	Nineteen	Green	Windows
Rachel	Has	Two	Cheap	Sofas
Steven	Kept	Fifteen	Pretty	Rings
Thomas	Ordered	Twelve	Red	Flowers
William	Wants	Sixty	White	Houses

Notes: Sentences were constructed by selecting one word from each of the five columns with equal probability, ensuring that transition probabilities between words were equated across sentences. Sentences were recorded in their entirety rather than as individual words.

Stimuli were presented using MATLAB (R2015a) and Psychtoolbox (version 3.0.14). Sounds were presented at 75 dB A, which was measured using a Brüel and Kjær (Nærum, Denmark) Type 2636 sound level meter. The levels were calibrated separately with the equipment that was used for the behavioral and MRI sessions.

### Experimental Procedures

#### Prescan Behavioral

At the beginning of the experiment, participants completed a behavioral session, to determine their thresholds for 60% and 90% performance on the SFG and SPIN tasks.

The prescan behavioral was conducted in a sound-attenuating booth. Participants sat in a comfortable chair facing an LCD visual display unit (Dell Inc.). Acoustic stimuli were presented through a Roland Edirol UA-4FX (Roland Corporation) USB soundcard connected to circumaural headphones (Sennheiser HD 380 Pro; Sennheiser electronic GmbH & Co. KG).

Participants first performed a short (<5 min) block to familiarize them with the figure-ground stimuli. During the familiarization block, they heard the figure and ground parts individually and together, with and without a gap in the figure.

After familiarization, we determined thresholds for the 2 tasks. We varied the TMR between the target (figure or speech) and masker (background tones or babble noise, respectively) in a weighted adaptive procedure (Kaernbach 1991). We used a step size ratio of 6:1 to estimate 60% thresholds and a step size ratio of 9:1 to estimate 90% thresholds. We used 4 separate blocks to estimate the TMRs corresponding to 60% and 90% thresholds for the two tasks. Each block included 2 separate but interleaved runs, which were identical except that different stimuli were presented. Each run started at a TMR of 0 dB and terminated after 10 reversals. The step size began at 1 dB and decreased to 0.5 dB after 3 reversals. Identical stimuli were used in the 60% and 90% blocks, but they were presented in different orders.

To estimate figure-ground thresholds, participants completed a yes-no task. On each trial, participants heard a figure-ground stimulus and had to decide whether or not there was a gap in the figure. The figure contained a gap on 50% of trials. On trials in which there was no gap in the figure, there was a gap in the background of the same magnitude (for details, see “Stimuli” section above). Participants responded by clicking buttons on the screen corresponding to yes and no responses.

During the SPIN blocks, participants also completed a yes-no task. They had to decide whether a sentence written on the screen was the same as the target sentence they heard spoken. The written sentence was presented on the screen after the spoken sentence had ended, and was identical to the spoken sentence on 50% of trials. It was different on 50% of trials: On these trials, one word in the written sentence differed from the word in the spoken sentence; this word occurred at each position in the sentence with equal probability, and was selected randomly from the other words in the corpus. Participants responded by clicking buttons on the screen corresponding to yes (same sentence) and no (different sentence) responses.

The order of the figure-ground and SPIN blocks were counterbalanced across participants. Before the first block of each task, participants performed a 6-trial practice at 3 dB TMR, with feedback.

#### Magnetic Resonance Imaging

The MRI session was completed on the same day, immediately after the prescan behavioral. The same figure-ground and SPIN tasks were presented, but at fixed TMRs—corresponding to the adapted TMRs from the prescan behavioral. Each task was presented at two different TMRs: One corresponding to the 90% threshold (SPIN-90 and SFG-90) and another corresponding to the 60% threshold (SPIN-60 and SFG-60).

Participants laid on a bed in the MRI scanner. Visual stimuli were presented through an Epson EB-L1100U projector, which participants viewed through a mirror attached to the head coil. Auditory stimuli were presented through a Roland Edirol UA-4FX (Roland Corporation, Shizuoka) USB soundcard connected to Ear-Tone Etymotic earphones (Etymotic Research, Inc.) with disposable foam ear tips.

**Figure 1:**
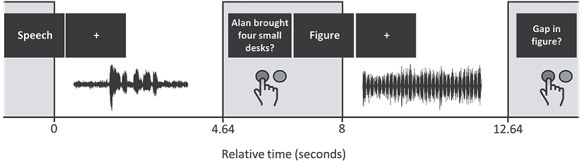
Schematic of the trial structure during the MRI session. The upper row displays the onset of visual stimulus presentation. The lower row displays the positioning of acoustic stimuli, and the approximate timing of button press responses. Gray bars represent functional volume acquisitions, which each lasted 3.36 s. Two exemplar trials are shown: a speech-in-noise trial followed by a figure-ground trial.

We presented 8 functional runs, each containing 24 trials. Each run contained 6 trials from each condition, which were pseudorandomly interleaved. Figure 1 shows a schematic of the trial structure. Each trial lasted 8 s and contained 3 major components: a visual cue, which indicated the task for the upcoming trial; the acoustic stimuli; and a probe sentence, which cued participants to make a response. We used a sparse sampling method with one functional MRI volume acquisition at the end of each trial. The acoustic stimuli were presented in the silent gap between scans. They began, on average, 1.4 s after the start of the trial and were jittered within an interval of 2 s (i.e., 0.4–2.4 s after the end of the previous scan). The visual cue was the word “figure” or “speech”; it was presented for 0.4 s (0.35 s during the previous scan, and 0.05 s after the previous scan had ended). A fixation cross then appeared on the screen until the probe sentence was presented 5.6 s after the trial began. The probe sentence remained on the screen until the visual cue for the next trial began. On figure-ground trials, the probe sentence was “Gap in figure?”. On SPIN trials, the probe was a written sentence followed by a question mark. Participants responded using a button box in their right hand; they pressed one button to respond “yes”—if the there was a gap in the figure (figure-ground task) or if the written sentence matched the spoken sentence (SPIN task)—and a different button to respond “no.”

Before participants began the MRI session, they first completed a practice of 24 trials outside the scanner using the same equipment that was used for the prescan behavioral. The trial structure of the practice was identical to the MRI session, and participants received no feedback about their responses.

### MRI Data Acquisition

MRI was conducted on a 3.0 Tesla Siemens MAGNETOM TIM Trio MR scanner (Siemens Healthcare) at the Wellcome Centre for Human Neuroimaging (London, UK) with a 64-channel receive coil.

T2^*^-weighted functional images were acquired using echo-planar imaging (EPI) with field of view of 192 × 192 × 144 mm; voxel size = 3.0 × 3.0 × 2.5 mm; echo spacing of 30 ms; time-to-repeat (TR) of 3.36 s; 48 slices; anterior-to-posterior phase encoding; bandwidth of 2298 Hz/Px. Acquisition was whole-brain transverse oblique, angled away from the eyes. We used sparse temporal sampling (Hall et al. 1999): A delay of 4.64 s was imposed between successive volumes, such that each volume acquisition began 8 s after the previous volume acquisition began. We collected 24 volumes from each participant in each of the 8 runs, plus an additional “dummy” scan, which was presented immediately prior to the first trial of each run and was excluded from the analyses. We collected field maps immediately after the functional runs (short TE = 10.00 ms and long TE = 12.46 ms).

At the end of the session, we acquired a whole-brain T1-weighted anatomical image (MPRAGE, 176 slices; voxel size = 1 mm isotropic; field of view 256 × 256 × 176 mm; PAT GRAPPA of factor 2; anterior-to-posterior phase encoding; TR = 2530 ms, TE = 3.34 ms).

### Analyses

#### Prescan Behavioral

We calculated thresholds as the median of the last 6 reversals in each run. We averaged the thresholds from the two interleaved runs within each block.

#### Behavior During Scan

To determine behavior during the MRI session, we calculated *d*′ (Green and Swets 1966) with loglinear correction (Hautus 1995). Trials with no response were included in the analysis as misses and false alarms (i.e., as incorrect responses).

#### MRI Preprocessing

MRI data were processed using SPM12 (Wellcome Centre for Human Neuroimaging). Each participant’s functional images (EPIs) were unwarped using their field maps and were realigned to the first image of the run. We then applied slice time correction. The functional and anatomical images were coregistered to the mean EPI, then normalized to the standard SPM12 template (avg305T1). We spatially smoothed the images using a Gaussian kernel with 4 mm full-width at half-maximum.

#### Statistical Parametric Mapping

Using SPM12, we modelled the fMRI timeseries for each participant with a General Linear (convolution) Model, with the motion realignment parameters as covariates of no interest. Each stimulus was modelled as a delta function, convolved with the canonical hemodynamic response function. We ran contrasts to test the main effect of Task (figure-ground and SPIN), the main effect of Difficulty (TMRs for 90% or 60% performance), and the interactions between Task and Difficulty. We entered the contrasts into a second level analysis, which applied one-sample *t*-tests at the group level. We examined these contrasts at the whole brain level, and applied appropriate family-wise error (FWE) correction to the results.

As a post-hoc (exploratory) analysis, we also applied small volume corrections over anatomical regions of interest: in bilateral auditory cortices, IFG, and IPS. We used standardized anatomical maps of auditory cortex (Morosan et al. 2001, 2005), which allowed us to locate activity across 4 subregions of auditory cortex: Te1.0, Te1.1, Te1.2, and Te3. Roughly speaking, activity in Te1.0 corresponds to core auditory cortex in medial Heschl’s gyrus, regions Te1.1 and Te1.2 to surrounding posteromedial and anterolateral belt areas, and Te3 to higher auditory cortex. We defined a bilateral auditory cortex ROI that included these subregions. For IFG, we defined a bilateral ROI encompassing BA 44 and BA 45 (Amunts et al. 1999), which cover the opercular and triangular parts of the IFG, respectively. For IPS, we defined a bilateral ROI containing hIP1, hIP2, and hIP3 (Choi et al. 2006; Scheperjans et al. 2008).

We also ran a contrast of all trials over the (implicit) baseline, which we used to define regions of interest (ROIs) for the DCM analysis.

#### Canonical Variate Analysis

We investigated multivariate activity within anatomical regions of interest using canonical variate analysis (CVA; Friston et al. 1996). This allowed us to test for effects that were distributed over voxels, with a greater sensitivity than equivalent mass univariate approaches. This was a post-hoc (exploratory) analysis designed to test whether the main effects of task, difficulty, or the interaction were evident in distributed responses over bilateral auditory cortex, IFG, or IPS. We used SPM12 to analyse each participant’s data, then we used Fisher’s method (Fisher 1925) to test for the significance of the ensuing canonical correlations at the group level.

#### Dynamic Causal Modeling

DCM is used to infer effective connectivity—and how directed causal influences among neural populations are affected by experimental manipulations. This distinguishes it from other methods for estimating functional connectivity that are based on correlations. In brief, neural dynamics are modelled by a bilinear differential equation. This equation includes the strength of connections between the modelled regions (the A matrix), the modulation of these connections as a function of experimental manipulations (e.g., changes due to task; the B matrix), and the strengths of direct inputs to the modelled system (e.g., sensory stimuli; the C matrix). The parameters correspond to rate constants. The model of neural population dynamics is combined with a hemodynamic model, which creates a joint forward model of the data. The joint model is inverted to give the posterior densities of the parameters. The technical details of DCM are explained in other papers (see Friston et al. 2003, 2017; Zeidman, Jafarian, Seghier, Litvak, Cagnan, Cathy, et al. 2019a; Zeidman, Jafarian, Seghier, Litvak, Cagnan, Price, et al. 2019b). We conducted the DCM analyses using SPM12. We first inferred the effective connectivity parameters that best fit each participant’s fMRI data, then estimated the parameters and their uncertainty at the group level using parametric empirical Bayes. Finally, we used a particular form of Bayesian model comparison—namely, Bayesian model reduction (BMR)—to establish which model of effective connectivity best explained the group data.

Regarding our sparse sampling design, DCM analyses have been successfully applied to sparse (auditory) fMRI data in previous work (Kumar, Stephan, et al. 2007b). From a modeling perspective, sparse acquisition is counterintuitively more efficient than conventional protocols because they preclude serial correlations in the data. In brief, the DCM predicts BOLD data at all time points, including those when no measurements were collected. Sparse acquisition ensures that data are conditionally independent of each other; thereby informing parameter estimates. Note that if the timeseries are not sampled efficiently then this would be apparent in the results of model comparison (e.g., with little difference in the evidence for two models).

Here, we were interested in making group-level inferences about how greater difficulty in figure-ground and SPIN tasks modulates intrinsic (i.e., within-region) and extrinsic (i.e., between-region) connectivity (i.e., a main effect of difficulty) and whether there are modulations specific to greater difficulty in one task over the other (i.e., an interaction between task and difficulty). To this aim, we used BMR to compare models that did and models that did not include modulations (of intrinsic and extrinsic connectivity) by the task–difficulty interaction. Greater evidence for models without modulations of intrinsic connectivity by the task–difficulty interaction provides evidence for shared processes between tasks.

##### Selection of Timeseries

We extracted timeseries for each subject in 8 ROIs (left and right Te1.0, Te1.1, Te1.2, and Te3). To ensure the timeseries showed reliable task-related activity—and did not include voxels with random signal fluctuations—we selected voxels for each subject that were significant at prespecified thresholds at both the group and individual-subject levels. In detail, we masked the group-level contrast (All trials > baseline) with anatomical masks extracted from the SPM Anatomy Toolbox (version 2.2c) (Eickhoff et al. 2005), corresponding to the 8 ROIs. We used the voxels that were below the *P* = 0.05 threshold (after FWE correction) to generate a functional mask for each ROI. We applied these functional masks to the individual-subject results, and retained voxels at the individual-subject level that were below a threshold of *P* = 0.05 uncorrected. Where no voxels within an ROI survived the *P* < 0.05 threshold (right Te1.0: 2/44 participants; right Te1.1: 11/44 participants; right Te1.2: 1/44 participants; left Te1.1: 2/44 participants; left TE1.2: 1/44 participants), we increased the individual-subject threshold in increments of 0.05 until one or more voxels survived. (Note that the thresholds applied in the selection of timeseries only specify the voxels that are included in the analysis and do not determine the probabilities identified by the DCM analysis.) Finally, we created a summary timeseries for each ROI in each participant by extracting the principal eigenvariate.

##### DCM Estimation

The DCM for each participant included 8 nodes corresponding to the extracted timeseries for the 8 ROIs. We specified the input timing for the DCM analysis as vectors specifying all trials of interest, the main effects of task and difficulty, the interaction between task and difficulty, and the motion and run covariates. We estimated (using Bayesian model inversion with variational Laplace) a fully connected DCM for each participant, which included all possible combinations of intrinsic and extrinsic fixed connections. We allowed all trials and the main effect of task to serve as external (i.e., direct or driving) inputs to each node. We allowed the main effect of difficulty and the interaction between task and difficulty to serve as external inputs to each node, and to modulate all intrinsic and extrinsic connections.

##### Group Level Inference

To estimate parameters at the group level (i.e., across participants), we took the parameters of interest for each participant to a second-level parametric empirical bayes (PEB; Friston et al. 2016) analysis. This is a hierarchical model of connectivity parameters, with connectivity parameters from all subjects at the first-level and a GLM at the second-level, estimated using a variational scheme. Our first-level parameters of interest were the modulations of intrinsic (within-region) and extrinsic (bottom-up and top-down) connectivity by the main effect of difficulty and by the task–difficulty interaction (i.e., the B matrix from each subject’s DCM). We entered the absolute TMRs in each of the 4 conditions for each participant (which differed according to their thresholds measured in the prescan behavioral session) as regressors in the second level of the PEB model. Having estimated parameters of the full PEB model, we then pruned away parameters using BMR—which performs an automatic (“greedy”) search over the model space, essentially comparing the evidence for reduced models that have particular parameters “switched off.” The model evidence considers both accuracy (how well the model fits the data) and complexity (the difference between model parameters and their prior values). Given we were interested in modulations of connectivity (i.e., the B matrix from each subject’s DCM), prior values for all parameters were set to zero. Parameters in DCM are rate constants and zero means that there was no effect of the experimental manipulation on the rate of change. In other words, the prior was that the modulations were “switched off,” and the model had to more accurately fit the data to justify the increase in complexity associated with switching the parameters “on.” The algorithm is given a model for which all parameters are “switched on,” and iteratively discards parameters if the reduced model has greater evidence. Thus, simpler models (i.e., those with more parameters “switched off”) that fit the data sufficiently accurately are preferred, because greater complexity reduces the model evidence. The final 256 models from the BMR were entered into a Bayesian model average (Hoeting et al. 1999; Penny et al. 2006), which performs a weighted average of parameters across models, according to the posterior probabilities (*P*_p_) of the models. This leads to a “winning model” that incorporates the uncertainty of parameter estimates. In practice, many of the 256 models entered into the analysis have low posterior probabilities, but this number ensures that all probable models will be captured in the Bayesian model average, and the uncertainty of the parameters estimated accordingly. We discounted parameters whose posterior probabilities were less than 0.95, to focus our conclusions on high-probability parameters; although, for completeness, we report the probabilities of all parameters in Table 4.

## Results

**Figure 2:**
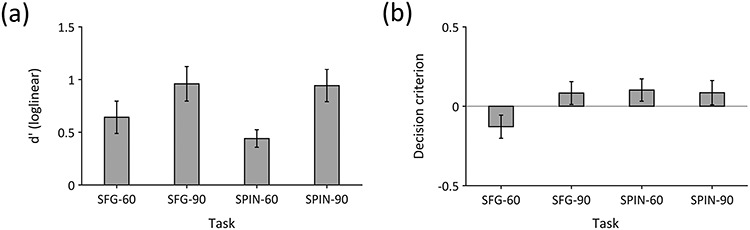
Behavior in the scanner in the SFG and SPIN tasks, at the two TMRs corresponding to 60% and 90% thresholds. Error bars display ±1 standard error of the mean. (*a*) Sensitivity. (*b*) Decision bias.

Behavior (*d*′) in the scanner followed the expected patterns (see Fig. 2). A two-way within-subjects ANOVA showed a significant main effect of difficulty [*F*(1,43) = 19.40, *P* < 0.001, *ω_p_*^2^ = 0.29] and no significant main effect of task [*F*(1,43) = 0.56, *P* = 0.46, *ω_p_*^2^ = −0.01]. The interaction between task and difficulty was not significant either [*F*(1,43) = 0.95, *P* = 0.34, *ω_p_*^2^ < 0.01]. These results confirm that the TMR (difficulty) manipulation was successful, and show that performance did not differ significantly between the 2 tasks.

**Table 2:** Contrasts between the SPIN and SFG tasks

Contrast	Peak location	*X* (mm)	*Y* (mm)	*Z* (mm)	*t*	*P_FWE_*
SPIN > SFG	Left STG	−63	−36	6	8.56	<0.001
		−60	−3	−3	8.00	<0.001
		−57	−12	0	7.97	<0.001
	Left precentral gyrus	−51	−12	42	7.95	<0.001
	Right STG	60	−21	0	7.48	<0.001
		66	−15	0	7.13	<0.001
		63	−6	−6	6.97	<0.001
	Right cerebellum	24	−60	−21	6.99	<0.001
	Right cerebellum	15	−66	−12	5.91	0.011
	Left inferior temporal gyrus	−45	−57	−9	5.82	0.014
	Left putamen	−30	−15	−3	5.61	0.029
	Left middle frontal gyrus	−39	6	36	5.55	0.036
	Cerebellar vermal lobules I–V	6	−57	−3	5.53	0.038
	Left STG	−51	−9	−12	5.52	0.040
	White matter	9	−75	24	5.47	0.047
SFG > SPIN	White matter	−21	−6	51	5.72	0.020
	Left superior parietal lobule	−36	−39	42	5.46	0.048

Notes: Statistical analyses were conducted at the group level using one-sample *t*-tests, and were thresholded at *P* = 0.05 after correcting for FWE. Peak locations were determined using Neuromophometrics (Neuromorphometrics, Inc.; http://neuromorphometrics.com/).

Figure 2 illustrates the corresponding results for decision bias: The bias was similar across conditions, but was lower in the SFG-60 condition. There was no significant main effect of task on decision bias [*F*(1,43) = 2.84, *P* = 0.10, *ω_p_*^2^ = 0.04]. However, due to lower bias in the SFG-60 condition, there was a main effect of threshold [*F*(1,43) = 8.57, *P* = 0.005, *ω_p_*^2^ = 0.14] and a significant interaction [*F*(1,43) = 10.16, *P* = 0.003, *ω_p_*^2^ = 0.17].

We also calculated Spearman’s rank correlation coefficients to investigate the relationship between thresholds, across subjects. Within each task, the 60% and 90% thresholds were significantly correlated (SPIN: *r_s_* = 0.51, *P* < 0.001, 95% confidence interval [CI] = 0.21–0.82; SFG: *r_s_* = 0.61, *P* < 0.001, 95% CI = 0.30–0.91). Thresholds were also significantly correlated between tasks (60% thresholds: *r_s_* = 0.36, *P* = 0.018, 95% CI = 0.05–0.66; 90% thresholds: *r_s_* = 0.31, *P* = 0.038, 95% CI = 0.01–0.62). This result confirms the behavioral relationship between figure-ground and SPIN performance, across subjects.

### Differences in Activity Between Figure-Ground and Speech-in-Noise Perception

**Figure 3:**
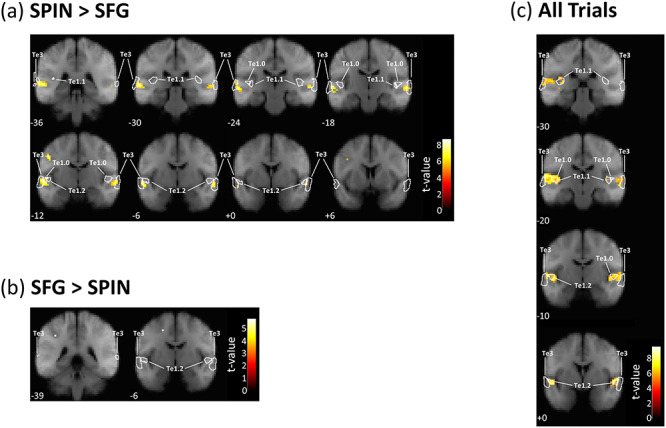
Group-level contrasts, thresholded at *P* < 0.05 (FWE corrected) and superimposed on coronal sections of the average (*N* = 44) T1-weighted structural image. (*a*) Voxels showing greater activity for the SPIN than SFG task. (*b*) Voxels showing greater activity for the SFG than SPIN task. (*c*) Voxels for the All Trials > baseline contrast. MNI co-ordinates (in mm) are displayed below each coronal section. White lines show the outlines of anatomical maps corresponding to areas Te1.0, Te1.1, Te1.2, and Te3.

The univariate BOLD analyses showed significant differences in activity between the two tasks. Table 2 lists the statistics and Figure 3*A*,*B* displays the thresholded SPMs. The SPIN task was associated with greater activity in bilateral STG, the left precentral gyrus, and the right cerebellum than the figure-ground task; these results all survived a threshold of *P* < 0.001 after FWE correction. At a threshold of *P_FWE_* < 0.05, the SPIN task was associated with greater activity in the left middle frontal gyrus, left inferior temporal gyrus, and left putamen. Whereas, the opposite contrast—greater activity for the figure-ground than SPIN task—yielded only two significant voxels, which survived a threshold of *P_FWE_* < 0.05 but not *P_FWE_* < 0.001. The finding that greater activity was revealed by the SPIN > SFG contrast than the SFG > SPIN contrast implies that the SPIN task activates more areas to a greater extent than the figure-ground task.

However, the univariate BOLD analysis did not show any effects of Difficulty. Neither the main effect of difficulty nor the task–difficulty interactions revealed activity below the *P_FWE_* = 0.05 threshold.

In addition to the whole brain analysis, we searched within predefined anatomical ROIs in auditory cortex, IFG, and IPS. The results of the ROI analyses were entirely consistent with the results at the whole brain level. For each ROI analysis, we applied a threshold of 0.05, corrected for FWE. We found significant activity in the auditory cortex ROI that was greater for SPIN than figure-ground trials. However, we found no significant voxels showing greater activity for figure-ground than SPIN trials. Within the same ROI, we found no significant voxels for the main effect of Difficulty or the Task–Difficulty interactions. We found no significant voxels in the IFG ROI for any of the contrasts. The only contrast that showed a significant effect in the IPS ROI was the contrast between figure-ground and SPIN trials, which identified greater activity in the figure-ground task in the same voxel as the whole brain analysis.

### Multivariate Activity is Sensitive to Differences between Figure-Ground and Speech-in-Noise Perception

The CVA analysis within the same anatomical ROIs showed that multivariate responses in all three regions (bilateral auditory cortex, bilateral IFG, and bilateral IPS) were sensitive to the contrast between the figure-ground and SPIN tasks (*X^2^* ≥ 640.43, *P* < 0.001). However, none of the regions’ multivariate responses were sensitive to the main effect of Difficulty (AC: *X^2^* = 77.91, *P* = 0.77; IFG: *X^2^* = 87.42, *P* = 0.50; IPS: *X^2^* = 97.32, *P* = 0.23) or the interaction between task and difficulty (AC: *X^2^* = 88.15, *P* = 0.48; IFG: *X^2^* = 80.12, *P* = 0.71; IPS: *X^2^* = 75.58, *P* = 0.82).

### Difficulty with Speech-in-Noise and Figure-Ground Perception Lead to Similar Disinhibition in Auditory Cortex

We used DCM to assess similarities and differences in effective connectivity. To inform the nodes for the DCM analysis, we contrasted all trials against baseline, which was orthogonal to our questions of interest, but identified regions activated by the four conditions. Table 3 lists the locations of voxels that survived the *P_FWE_* < 0.05 threshold, and Figure 3C shows the locations on four coronal slices. The voxels that were most reliably activated by the tasks (which survived a correction of *P_FWE_* < 0.001) were located on the superior temporal lobe—with peaks in the left transverse temporal gyrus (Heschl’s gyrus), left planum temporale, and bilateral planum polare.

**Table 3:** Contrast of all tasks > baseline

Peak location	*X* (mm)	*Y* (mm)	*Z* (mm)	*t*	*P_FWE_*
Left planum temporale	−60	−18	9	9.55	<0.001
Left transverse temporal gyrus	−51	−15	6	9.45	<0.001
Left planum polare	−45	−12	0	8.75	<0.001
Right planum polare	57	3	0	9.01	<0.001
Right cerebral white matter	63	−12	3	8.89	<0.001
Right planum polare	51	−3	−3	7.91	<0.001
Right anterior insula	30	24	0	6.74	0.001
Right frontal operculum	33	18	9	5.60	0.030
Left supplementary motor cortex	−6	18	45	6.31	0.003
Left opercular inferior frontal gyrus	−48	21	24	5.86	0.013
Left superior parietal lobule	−27	−54	48	5.60	0.031
Left anterior insula	−39	18	−3	5.58	0.032
Left anterior insula	−33	21	0	5.58	0.032
Right planum polare	42	−15	−6	5.55	0.036
Left cerebral white matter	−39	−30	0	5.55	0.036
Right cerebellum	21	−66	−51	5.52	0.040
Right opercular inferior frontal gyrus	48	9	21	5.48	0.045
Right opercular inferior frontal gyrus	57	21	15	5.48	0.045

Notes: Statistical analyses were conducted at the group level using one-sample *t*-tests, and were thresholded at *P* = 0.05 after correcting for FWE. Peak locations were determined using Neuromophometrics (Neuromorphometrics, Inc.; http://neuromorphometrics.com/).

Given DCM works best with ≤8 nodes and for areas that are reliably activated by a task, we focussed broadly on these parts of auditory cortex as areas of interest for the DCM analyses. Although some regions outside of auditory cortex survived the *P_FWE_* < 0.05 threshold (see Table 3), these were not as strongly activated at the group level, and were not reliably identifiable in individual subjects. Notably, the IPS was not present at the group level, even with the *P_FWE_* < 0.05 threshold—therefore, we did not include this region in the DCM analysis. The choice to focus on areas of auditory cortex allowed us to address hypotheses about intrinsic (within-region) and extrinsic (top-down and bottom-up) connectivity within a set of nodes (i.e., a subgraph) where we observed reliable activity. It is worth noting that external (i.e., direct) connections were allowed to influence all nodes, meaning that the model accommodates activity that is better explained by direct inputs (e.g., including regions outside those that we explicitly modelled) than connectivity between the modelled nodes.

To parcellate the functional activity into separate subregions of auditory cortex, we used standardized anatomical maps (Morosan et al. 2001, 2005), which allowed us to separate activity in 8 subregions, corresponding to bilateral Te1.0, Te1.1, Te1.2, and Te3. Functional activity in these 8 subregions was used for the DCM analysis.

We used BMR and averaging to identify the winning DCM model, which is defined as the model with the greatest evidence among models with all possible combinations of parameters. These parameters quantified the modulatory effects of difficulty and the interaction between task and difficulty on each connection. The model included both intrinsic (i.e., self) connections and extrinsic (bottom-up and top-down between nodes) connections. In DCM, the parameters are rate constants, which control the rate of exponential decay of neuronal activity during and after stimulation. At the group level, we included the absolute TMR values for each participant as between-subject regressors.

**Figure 4:**
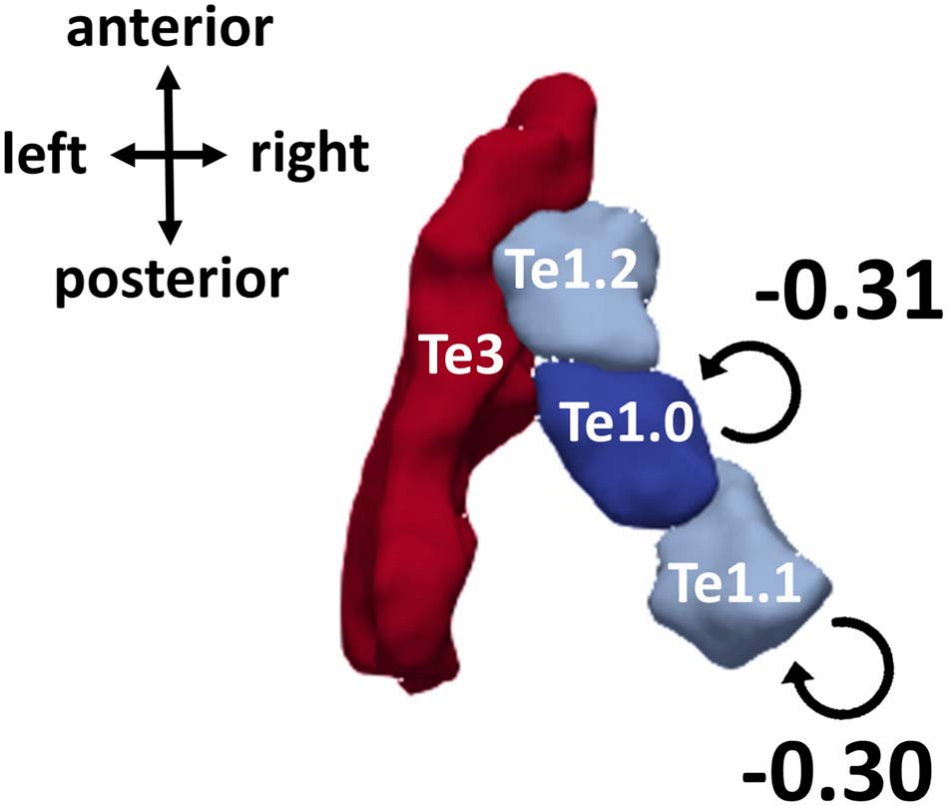
Group-level DCM, after BMR and averaging. Parameters that survive a threshold of posterior probability (Pp) > 0.95 are displayed: These correspond to modulations of intrinsic connectivity by the main effect of difficulty in left Te1.0 and 1.1. Their connection strengths (in Hz) are displayed in the figure. Results are displayed on a 3D reconstructed surface of the anatomical regions of interest in the left hemisphere, which was generated using ITK-SNAP (www.itksnap.org; Yushkevich et al. 2016) and ParaView (www.paraview.org; Ahrens et al. 2005). Note that right-hemisphere homologs were included in the DCM, but none of the parameters had posterior probabilities greater than 0.95. Extrinsic (top-down and bottom-up) connections were also included, but modulations of these connections were absent in the winning model.

Table 4 displays the parameters in the winning group-level DCM. Essentially, this means that other models—that contain either more or fewer parameters—are less likely (given the fMRI data) than the winning model. Only two of the parameters in the winning DCM had high probabilities (*P*_p_ > 0.95). These two parameters correspond to the modulation of intrinsic (i.e., self) connections for left Te1.0 and left Te1.1 by the main effect of difficulty (Fig. 4). The values of these parameters were −0.31 and −0.30 Hz, respectively, which indicate a decrease in self-inhibition associated with the experimental effects.

**Table 4:** Parameters in the DCM after BMR and averaging

Parameter	Connection strength	Posterior probability
**Commonalities**		
Difficulty modulation: Left Te1.0 (intrinsic)	−0.47	>0.99
Difficulty modulation: Left Te1.1 (intrinsic)	−0.51	>0.99
*Difficulty modulation: Left Te1.3 (intrinsic)*	*−0.26*	*0.94*
*Difficulty modulation: Right Te1.1 (intrinsic)*	*−0.18*	*0.71*
***SFG-60 TMR***		
*Interaction modulation: Left Te1.1-Left Te1.2*	*−0.02*	*0.78*
***SPIN-60 TMR***		
*Difficulty modulation: Left Te1.2 (intrinsic)*	*−0.05*	*0.76*
*Difficulty modulation: Right Te1.0 (intrinsic)*	*−0.05*	*0.73*
*Difficulty modulation: Right Te1.2 (intrinsic)*	*−0.06*	*0.81*

Notes: TMR in each of the four conditions (SPIN-90, SPIN-60, SFG-90, SFG-60) were included as regressors. The connection strengths and posterior probabilities associated with the parameters (based on the free energy with and without the parameter) are displayed in the final columns. Parameters with probabilities <0.95 are listed in *italics.*

Interestingly, all of the parameters corresponding to modulations of connectivity by the task–difficulty interaction were pruned away, as were as all of the modulations of extrinsic (bottom-up and top-down) connectivity—and therefore, these parameters are not present in the final model. In other words, the evidence was greater for models that had these connections “switched off” than models that had them “switched on.” Thus, this model comparison provides very strong evidence that, in auditory cortex, effects of difficulty on effective connectivity are shared between the SPIN and figure-ground tasks. The model comparison does not support partially shared effects of difficulty (which would be indicated by modulations of effective connectivity by both difficulty and the task–difficulty interaction in the winning DCM) or separate effects (which would be indicated by modulations of effective connectivity by the task–difficulty interaction only in the winning DCM).

Some of the model parameters covaried with the TMR in one of the four conditions, although it is worth noting that their probabilities were low (≤0.81; Table 4).

## Discussion

Our results demonstrate that SPIN and figure-ground perception rely on similar cortical processes. Using DCM, we found that greater difficulty in both tasks leads to similar disinhibition at lower levels of the auditory cortical hierarchy: We found strong evidence (>99% probability) that left Te1.0 and left Te1.1 are disinhibited when the tasks are more difficult—indicating that these regions likely increase their gain when speech or figures are difficult to follow. Importantly, we found the best model of the data had no modulations of auditory cortex activity by the task–difficulty interaction, showing that within auditory cortex the evidence was greater for models that had only common connections between the two tasks than for models that included task-specific connections. This result demonstrates that effective connectivity in auditory cortex is common to the two tasks (and possibly other tasks that engage similar grouping processes), rather than task-specific. Overall, these results provide evidence for a common cortical substrate in early auditory cortex, which informs current models of early sensory processing and could explain why people who find SPIN perception difficult also find figure-ground perception difficult (Holmes and Griffiths 2019).

The results showed that lower level intrinsic connectivity, rather than top-down connectivity from higher to lower subregions of auditory cortex, was modulated by task difficulty—consistent with previous reports that activity in early auditory cortex is modulated by attentional load during figure-ground analysis (Molloy et al. 2019). Consistent with these results, a previous MEG study (Giani et al. 2015) found that detecting pairs of same-frequency tones against a background was associated with intrinsic connectivity changes in auditory cortex—although, subregions of auditory cortex were not examined. Their paradigm differed from ours in that the tones were longer (300 ms), the pairs of tones to be detected were separated in time (by 750 ms), and the target tones were in a different frequency region to background tones and could therefore be detected by frequency alone. Previous studies of visual scene analysis that used DCM similarly found that lower level intrinsic connections, rather than top-down connections, offered the best explanation for modulations of BOLD activity by the “noisiness” of a random dot motion stimulus (Adams et al. 2015, 2016). Intrinsic connections in DCM are rate constants, which control the rate of decay in a region, and disinhibitory modulations indicate slower decay. In other words, disinhibition indicates that an area becomes more excitable. In predictive coding formulations of perceptual synthesis, this is usually interpreted in terms of assigning more precision to prediction errors, so that they have greater influence on evidence accumulation or Bayesian belief updating. From a psychological perspective, this is usually thought of in terms of attentional selection (Brown et al. 2013; Bauer et al. 2014; Vossel et al. 2014, 2015; Auksztulewicz and Friston 2015; Kanai et al. 2015).

A likely mechanism for the apparent disinhibition of intrinsic connections in the DCM is increased neuronal gain or excitability (Reynolds and Heeger 2009), which is well-established in auditory cortex (Rabinowitz et al. 2011). This could be realized by communication through coherence, by spiking interinhibitory neurons equipped with *N*-methyl-d-aspartate (NMDA) receptors, or by modulations through acetylcholine (ACh). Broadly speaking, these mechanisms are unlikely to be synapse-specific and would therefore more likely manifest in changes in intrinsic than extrinsic connectivity. Modulations of particular extrinsic (e.g., top-down) connections would be more consistent with synapse-specific effects.

Modulations of lower level intrinsic connectivity are also consistent with the results of some previous studies examining speech perception. For example, Mattys et al. (2005) found that when speech is accompanied by white noise, lexical decisions are based on lower level (word stress) cues rather than higher level (e.g., context) cues. In addition, several studies have proposed that listening challenges during speech perception are realized by feedforward rather than feedback processes. For example, Davis et al. (2011) used fMRI to measure responses to spoken sentences. They found that listening challenges introduced by presenting speech at a low TMR or by presenting semantically ambiguous sentences were associated with early responses in anterior STG, which preceded later changes in higher areas. They interpret their results as reflecting a greater demand on internal representations of unanalysed speech when speech perception is challenging. Using DCM, Leff et al. (2008) found that feedforward, rather than feedback, connections were associated with the difference between intelligible speech in quiet and unintelligible (time-reversed) speech. In addition, these lower level regions have previously been associated with speech intelligibility in some studies: Binder et al. (2004) found that an anteriolateral temporal region—corresponding to Te1.1—correlated with accuracy on a phoneme-in-noise discrimination task, and Wild et al. (2012) found that activity in Te1.0 covaried with the intelligibility of degraded speech. The current results support the idea that lower level processes (in Te1.0 and Te1.1) are associated with the perceptual challenge of a less favorable TMR during SPIN perception. In addition, we show that perceptual challenges during figure-ground perception affect these lower level processes in a similar way as do challenges during SPIN perception.

A previous fMRI study of figure-ground perception (Teki et al. 2011), which used the same maps of auditory cortex that we used here, found no evidence for activity associated with figure salience in primary auditory cortex—although they did not use a task, whereas we used a relevant, active task. A previous EEG study (O’Sullivan et al. 2015) found greater activity during active than passive listening to figure-ground stimuli, and a MEG study (Molloy et al. 2019) similarly found greater activity in primary auditory cortex under low than high visual load. Thus, our results are consistent with the idea that task effects modulate early auditory cortex during figure-ground perception. Here, we extend this idea by showing that the earliest stages of the auditory cortical hierarchy are more engaged (i.e., less inhibited) when the figure-ground task is more challenging due to a lower TMR.

Studies using other simultaneous or sequential stream segregation to study perceptual organization have found that activity in both primary (Fishman et al. 2001; Micheyl et al. 2005; Bidet-Caulet et al. 2007; Wilson et al. 2007; Deike et al. 2010; Schadwinkel and Gutschalk 2010) and nonprimary (Wilson et al. 2007; Deike et al. 2010; Schadwinkel and Gutschalk 2010) auditory cortex differs depending on how listeners perceive acoustic sources—for example the number of sources they perceive or which features of the scene they attend to. Also, Overath et al. (2010) found both primary and nonprimary parts of auditory cortex were active when participants detected changes in spectrotemporal coherence in dense acoustic “textures,” which contain multiple components that changed frequency; participants made decisions about the how coherent the direction of frequency changes were across components.

We did not find reliable activity affected by difficulty in higher areas such as IPS: thus, we did not model these high-level responses using DCM. In earlier work, IPS activity has been attributed to top-down attention (Cusack 2005) or to perceptual “pop-out” (Shamma and Micheyl 2010; Teki et al. 2011) during auditory scene analysis. Although inferior parietal activity has been associated with both figure-ground perception (Teki et al. 2011, 2016) and SPIN perception (Alain et al. 2018), the co-ordinates in left IPS reported for figure-ground perception (Teki et al. 2011) are either more superior or more posterior and medial to the peak co-ordinates reported by Alain et al. (2018). This could explain why we found no reliable inferior parietal activity in the current study, and is consistent with the differences we found in IPS between the SPIN and figure-ground tasks. In addition, we were looking for differences between 60% and 90% performance levels, which are not as distinct as in some previous studies where, in the easiest conditions, participants perform comfortably at ceiling level. In the current study, it was important to equate performance levels between the two tasks and, therefore, we targeted performance below ceiling levels. Under these conditions, we found the strongest and most consistent effects in auditory cortex. We did find two voxels showing greater activity for figure-ground than SPIN perception located close to parts of the IPS that have previously been associated with figure-ground perception (Teki et al. 2011, 2016)—but the two voxels were spatially separate and did not survive a stringent correction for FWE at an alpha of 0.001. It is likely that effects of difficulty that are specific to figure-ground and SPIN perception occur at higher levels of the auditory pathway. Here, our aim was to search for commonalities between these 2 tasks—reflecting common grouping processes—for which we found strong evidence in early auditory cortex.

The strongest—and highest probability—modulations of effective connectivity by difficulty were located in the left hemisphere; although, it is worth bearing in mind that it was not an aim of this experiment to test for laterality effects. It is widely accepted that speech is processed bilaterally in auditory cortex (see Peelle 2012; Scott and McGettigan 2013). However, the left hemisphere modulations we observed are consistent with previous studies that have localized SPIN effects to the left hemisphere. For example, Scott et al. (2004) found an area within the left anterior STG that showed a positive correlation with speech intelligibility, and Davis et al. (2011) found that the left posterior STG correlated positively with TMR. Regarding more basic stimuli designed to assess auditory scene analysis, two previous studies of sequential stream segregation found correlates of different percepts in the left but not right auditory cortex (Deike et al. 2004, 2010). Flinker et al. (2019) suggest that perceiving temporal modulations leads to left lateralised responses, whereas perceiving spectral modulations leads to right lateralised responses. Unlike other figure-ground tasks (Kidd 1994; Kidd et al. 1995; Gutschalk et al. 2008; Elhilali, Xiang, et al. 2009b), the SFG task used here cannot be detected based on simple spectral separation; instead, it has been associated with a temporal coherence mechanism (Shamma et al. 2011; Teki et al. 2013). That we found predominantly left-hemisphere modulations therefore aligns both with the division proposed by Flinker et al. (2019), and with the findings from previous SPIN tasks. However, it is worth noting that one right hemisphere intrinsic connection (Te1.1) was present in our DCM (Table 4), albeit with a lower probability (0.71)—suggesting the effects are not entirely lateralised. One previous EEG study (Bidelman and Howell 2016) reported greater right-hemisphere lateralisation when SPIN was presented at a lower TMR, although in that study, participants were instructed to ignore the speech stimuli—and, therefore, responses are unlikely to relate to poorer intelligibility and may instead relate to stimulus acoustics.

In the current study, we manipulated difficulty by manipulating TMR, which is a naturally relevant quantity that varies greatly among different everyday listening settings. Rather than specifying a TMR that was fixed across participants, we selected TMRs for each participant that corresponded to 60% and 90% behavioral thresholds. This aspect of the design makes it less likely that the results reflect acoustic properties of different TMRs, but rather the perceptual challenges imposed by a lower TMR—which occur at different TMRs for different people. The selected TMRs and the acoustic noise (babble for SPIN; random tone chords for figure-ground) also differed between the two tasks. Furthermore, absolute TMRs in each participant were regressed out of the model. Therefore, disinhibition of left Te1.0 and Te1.1 likely arose due to the increased difficulty associated with lower TMRs, rather than acoustic properties of the SPIN and figure-ground tasks that covary with TMR.

Given we compared only two tasks, one might ask whether disinhibitory processes in primary auditory cortex are common to all challenging auditory tasks. Crucially, the behavioral correlation between figure-ground and SPIN thresholds reported by Holmes and Griffiths (2019)—which we replicated here—was specific to particular types of figures: It explained a different portion of the variance in SPIN performance than did a figure that changed frequency over time, and no relationship was present for a more complex figure whose components changed frequency independently. Thus, there is clearly high specificity in the relationship between figure-ground and SPIN perception. Second, our results likely reveal something more than basic acoustic properties: the 2 tasks used different stimuli with different spectrotemporal characteristics and cannot be attributed to the absolute TMRs we used, which differed across participants and were regressed out of the model.

Possibly, between-subjects differences in the disinhibition of left Te1.0 and 1.1 might help to explain the behavioral correlation between figure-ground perception and SPIN perception—which was reported by Holmes and Griffiths (2019) and replicated here. In other words, some of the individual variability in SPIN perception may arise from differences in disinhibition at the early stages of auditory cortical processing related to the grouping of speech segments in background noise. Given our figure-ground task taps into similar cortical processes, this could be a useful nonlinguistic test for assessing changes in cortical disinhibition that might explain why some patients report difficulties with SPIN perception, despite no evidence of impaired peripheral function (Cooper and Gates 1991; Kumar, Amen, et al. 2007a; Hind et al. 2011). Our univariate analyses indicate that figure-ground perception predominantly activates a subset of the regions involved in SPIN perception, consistent with the idea that our figure-ground task is a basic version of SPIN perception that relies on similar acoustic analysis (e.g., fundamental grouping processes), but does not require linguistic and articulatory processes involved in SPIN perception. The current experiment was not designed to pursue this between-subjects question, given the close matching of behavioral performance between the tasks during the fMRI session, but it would be an interesting direction for future research.

The results of the univariate analysis indicate that figure-ground perception predominantly activates a subset of the regions involved in SPIN perception: both tasks reliably activate the superior temporal lobe (Table 3), but SPIN perception leads to greater activity in bilateral STG, the left precentral gyrus, and the right cerebellum (Table 2). This finding is consistent with the idea that our figure-ground task is a basic version of SPIN perception that relies on similar acoustic analysis (e.g., fundamental grouping processes), but does not require linguistic and articulatory processes involved in SPIN perception.

The areas that were more strongly activated by SPIN than figure-ground—bilateral STG, the left precentral gyrus, and the right cerebellum—have been associated with SPIN tasks in previous studies. For example, the STG correlates positively with speech intelligibility (Scott et al. 2004) and TMR (Davis et al. 2011). Cerebellar activity has also been reported in previous studies (see Ackermann et al. 2007 for a review), despite the fact that—traditionally—the cerebellum is not commonly thought to be part of the speech network. Using PET, Salvi et al. (2002) found activity in the right cerebellum when speech-in-babble was compared with speech-in-quiet; perhaps, crucially, they selected levels for the speech and babble to ensure that performance was approximately 50% for each participant. Similarly, here, we ensured that speech intelligibility was below ceiling (60% or 90%) and equated performance between the SPIN and figure-ground tasks. The involvement of the motor cortex (precentral gyrus) in speech perception has been long debated (see Scott et al. 2014), but several studies have found motor cortex responses during speech perception (e.g., Wilson et al. 2004; Wilson and Iacoboni 2006). Areas for speech production are thought to be particularly important for comprehending degraded speech (e.g., Hervais-Adelman et al. 2012). The current results lend further support to the claim that, compared with elementary auditory stimuli, challenging speech perception engages motor cortex.

Only two voxels showed greater activity for figure-ground than SPIN perception, and they did not survive a stringent correction for FWE at an alpha of 0.001. However, it is worth noting that they are located close to parts of the IPS that have previously been associated with figure-ground perception (Teki et al. 2011, 2016). Consistent with these results, earlier work has demonstrated that IPS plays a role in basic auditory streaming (Cusack 2005). Previously, IPS activity has been attributed to top-down attention (Cusack 2005) or to perceptual “pop-out” (Shamma and Micheyl 2010; Teki et al. 2011) during auditory scene analysis. During figure-ground perception, predictions about frequencies can be very precise after the figure has been detected (because the frequencies of the figure remain the same for the entire figure duration), whereas speech changes frequency over time; thus, greater activity in IPS during figure-ground perception could reflect greater “pop-out” of figures that remain the same frequency over time, than of speech, which changes frequency over time.

We did not find any voxels to be reliably activated by the main effect of difficulty in our univariate analysis. We also found no evidence that multivariate activity in bilateral auditory cortex, IFG, and IPS was sensitive to the main effect of difficulty. Although we might have found frontal or parietal activity, this did not emerge strongly in our analyses. Unlike previous studies, this study examined consistent activity between figure-ground and SPIN tasks, which might explain why areas beyond auditory cortex did not appear strongly. In addition, it is worth noting that the levels of difficulty that we chose (60% and 90% thresholds) are not as distinct as in some previous studies where, in the easiest conditions, participants perform comfortably at ceiling level. It is possible that areas beyond auditory cortex might be reliably activated under a greater difference in task difficulty. However, in the current study, it was important to equate performance levels between the two tasks and, therefore, we targeted performance below ceiling levels. Under these conditions, we found the strongest and most consistent effects in auditory cortex.

In summary, our results demonstrate common processes for figure-ground and SPIN perception in early auditory cortex. We found that figure-ground perception predominantly activates a subset of regions involved in SPIN perception. Modeling of BOLD responses showed that greater difficulty in both tasks is associated with disinhibition in left Te1.0 and left Te1.1—implying that the early stages of the auditory cortical hierarchy increase their gain when SPIN and figure-ground perception become more difficult. Ultimately, these results suggest a common cortical substrate that links perception of basic and natural sounds—and might explain why people who are worse at figure-ground perception are also worse at SPIN perception.

## Notes

We would like to thank Stuart Rosen, who provided the 16-talker babble track that we used in this experiment. *Conflict of Interest:* The authors declare no competing interests.
